# Why Is Norfolk Sickly?

**Published:** 1855-10

**Authors:** 


" *Why is Norfolk Sickly?*"—All our readers have doubtless heard of the unprecedented ravages of the Yellow Fever during the latter part of the summer in Norfolk and Portsmouth, Va. They will be glad to know that the disease has now nearly ceased its ravages for this season. The following answer to the question which constitutes our caption, we copy from the Boston *Medical and Surgical Journal:*

"Under the above head, we notice, in a number of the N. Y. *Daily Tribune*, an article descriptive of the locality of this ill-fated city, which justifies the words with which this article commences,—"A more reasonable question to ask would be, why is it ever healthy?" The following extract gives an idea of the situation of Norfolk and Portsmouth.

"The two towns are situated on an arm of the sea projecting far inland from Chesapeake Bay. * * * * All the adjacent lands have so recently emerged from the sea, that the mud and malaria-generating matter have not yet undergone the change necessary to make it healthy for the abode of men. * * * * The Dismal Swamp, one of the largest, deepest and most impenetrable in the United States, almost touches the suburbs of Portsmouth, lying on the south side of one of the little arms of the bay; while Norfolk is situated opposite, on the north, surrounded on three sides with stagnant water; immediately in contact with the houses, and all around, for twenty miles or more, the land is almost flat and but slightly elevated above the sea level."

This description is confirmed by a resident for the past two years in Norfolk, who informs us that the city is destitute of sewerage, and that its streets are extremely filthy, being often strewed with refuse vegetables and other garbage, which result from the immense quantity of provisions brought into the city, for export. These matters become rotten, and emit a most noisome stench. The turkey-buzzard, the natural scavenger of the South, is not found in Norfolk, but his place is supplied by cows, who wander at will through the town, and gather an unhealthy subsistence from the cabbage stalks and other substances which lie in heaps on the ground. The condition of Portsmouth is much worse than that of Norfolk. It is connected with Gosport by a causeway, nearly a mile in length, if we are not mistaken, across a swamp or flats, from which arises a powerful stench.

These facts are sufficient to account for the extraordinary spread of the yellow fever, after its first introduction, and we trust they will be so far appreciated by the local authorities as to lead to the institution of sanitary improvements as soon as the subsidence of the epidemic shall allow the necessary measures to be taken. It may not be possible to prevent the disease from re-appearing, but cleanliness and drainage will go far towards restraining the scourge within comparatively moderate limits. In the meantime, let us hope that the lesson may not be lost upon other places in a similiar hygienic condition,—that the apathy which exists so extensively on this important subject may be exchanged for an enlightened wisdom in sanitary reform,—that the maxim, "cleanliness is next to godliness," may become a proverb throughout our land.

